# Treatment of Hepatitis B in Decompensated Liver Cirrhosis

**DOI:** 10.4061/2011/918017

**Published:** 2011-06-23

**Authors:** Richard Guan, Hock Foong Lui

**Affiliations:** ^1^Mount Elizabeth Hospital and Medical Centre, Singapore 228510; ^2^Gleneagles Hospital and Medical Centre, Singapore 258500

## Abstract

Chronic hepatitis B infection progresses from an asymptomatic persistently infected state to chronic hepatitis, cirrhosis, decompensated liver disease, and/or hepatocellular carcinoma. About 3% of patients with chronic hepatitis develop cirrhosis yearly, and about 5% of individuals with hepatitis B cirrhosis become decompensated annually. The outcome for patients with decompensated cirrhosis is bleak. Lamivudine, the first oral antiviral agent available for hepatitis B treatment is safe and effective and can improve or stabilize liver disease in patients with advanced cirrhosis and viraemia. Viral resistance restricts its prolonged use. Entecavir and tenofovir are newer agents with excellent resistance profile to date. These and some other antiviral agents are being investigated for optimal use in this rather challenging patient group.

## 1. Introduction

Chronic hepatitis B virus (HBV) infection is a major global public health problem with an estimated 1 million deaths yearly worldwide from complications of liver cirrhosis namely, liver failure and hepatocellular cancer (HCC) [1, 2]. Up to 40% patients with chronic hepatitis B virus infection develop serious complications during their lifetime. Up to 12% of patients with HBV cirrhosis die of liver failure, and up to 10% perish from liver cancer [3, 4]. The prognosis for patients with decompensated HBV cirrhosis is poor, with a 5-year survival of only 14% compared with 84% in patients with compensated HBV cirrhosis [3]. The ultimate cure for end stage liver disease is liver transplantation. Many patients with advanced hepatitis B worldwide do not have access to or are not eligible for this treatment modality [1, 5]. This article briefly review the epidemiology and natural progression of chronic hepatitis B infection and provides an update on the medical management of patients with decompensated HBV cirrhosis with particular emphasis on the use of available antiviral agents. 

## 2. Prevalence of Hepatitis B Infection

Hepatitis B virus (HBV) infection is endemic in the Asia Pacific region and in Africa. Up to 62% of the population in China, up to 98% of the people in sub-Saharan Africa and up to 80% of the populations in some Pacific islands have markers of HBV infection [6]. Chronic or persistent HBV infection is defined as the presence of hepatitis B surface antigen (HBsAg) in the serum for longer than 6 months. It is estimated that there are 350 million people with chronic HBV infection worldwide (more than 5% of the world population). More than 75% of these chronically infected people live in Asia and a further 12%, (approximately 50 million) live in Africa. In many countries in the Asia Pacific region, 8–20% of the populations have chronic HBV infection [7]. 

## 3. Disease Progression in Chronic Hepatitis B Infection

Chronic HBV infection can lead to chronic liver disease, with a broad range of symptoms [8, 9]. The early phase of the infection is typically asymptomatic with active virus replication (HBVDNA > 20,000 IU/mL) and very little liver necroinflammation. Hepatitis B antigen (HBeAg) is present. This phase, also called the immune-tolerant phase, can last for several decades before the appearance of hepatitis symptoms. Persistent or episodic hepatic necroinflammation (chronic hepatitis), with elevated serum alanine aminotransferase (ALT) levels, characterizes this phase of immune elimination of HBV. During this stage, HBeAg and HBV DNA levels may progressively decrease and seroconversion from HBeAg to HBe antibody (anti-HBe) may occur [10, 11]. The longer this period of active liver disease lasts, the higher the risk of irreversible liver damage. Patients enter the third (inactive carrier) phase when HBV replication is no longer detected and liver histology usually stabilizes [10]. However low levels of the virus (HBVDNA < 2000 IU/mL) can stillbe detected in the majority of patients, and reactivation of HBV replication with exacerbation of disease can occur [12–14]. Some patients may progress to cirrhosis and HCC during this phase of apparent inactivity. 

In the Asia-Pacific region, the annual incidence of chronic hepatitis in asymptomatic, persistently infected individuals ranged from 0.84 to 2.7%. The annual incidence of cirrhosis among patients with chronic hepatitis B was reported to be approximately 1.0–2.4%. The annual rates of progression from compensated cirrhosis to decompensated cirrhosis were around 4.6% [15, 16]. The annual incidence of HCC ranged from 0.02–0.65% in asymptomatic persistently infected individuals, 0.27–1.0% in patients with chronic hepatitis B and 3.0–6.6% in compensated HBV cirrhosis [6]. The annual progression rate from decompensated HBV cirrhosis to HCC was around 7.1%. The 5-year mortality rate from decompensated cirrhosis was from 41% to 67% [17, 18].

Factors associated with rapid disease progression in HBV infected patients include the male gender, increasing age, viraemia with repeated hepatic flares or prolonged periods of liver necroinflammation, and alcohol use; confection with other viruses such as hepatitis C, hepatitis D, and human immunodeficiency virus (HIV); use of immunosuppressive agents, platelets less than 150,000/mL and serum bilirubin more than 1.1 mg/dL (18.8 umol/L) [19–26]. Patients with HBV cirrhosis and active viral replication are at increased risk of developing progressive liver disease and death [4, 27]. Loss of HBeAg and seroconversion to HBe antibody (anti-HBe) with reduction in HBVDNA levels have been associated with a 55% reduction in the risk of death [3]. Suppression of HBV replication with loss of HBeAg and or HBsAg is therefore an important event in the natural history and treatment of chronic HBV infection. 

## 4. Liver Cirrhosis and Decompensation

The mean age of onset of cirrhosis in chronic HBV infection acquired during childhood, is about 40 years and complications become clinically evident 3 to 5 years later. It is estimated that the annual rate of hepatic decompensation is 4% in cirrhotic patients with viraemia and 1% in those without viraemia [14]. The development of jaundice, ascites, hepatic encephalopathy (HE) or bleeding oesophageal varices signals decompensation. Acute decompensation is usually secondary to a hepatitis flare or spontaneous bacterial infection which further impairs the already decreased hepatic reserve. The other form is a gradually developing end-stage event. As mentioned previously, the outlook for decompensated cirrhosis is rather bleak with a 5-year survival of 14% compared with 84% in patients with compensated cirrhosis [3]. 

## 5. General Management of Patients with Decompensated Liver Cirrhosis

### 5.1. Assessment of Disease Severity

Clinical examination and measurement of blood parameters like serum albumin, bilirubin, creatinine and prothrombin time can help determine the severity and progression of liver disease.The Child-Turcotte-Pugh (CTP) score and Model for End Stage Liver Disease (MELD) score are two indices that are usually used to determine the severity of liver disease in patients with cirrhosis [28] (Table 1). The CTP score was developed to determine preoperative risk of patients with cirrhosis for portal-systemic shunt surgery. It is calculated by adding the individual scores assigned to ranges of serum albumin level, serumbilirubin level, prothrombin time, the presence and degree of ascites and hepatic encephalopathy. The CTP score is easily calculated at the patient's bedside. The MELD score was initially developed to predict short-term mortality following transjugular intrahepatic portosystemic shunt (TIPS) placement. It was later modified to predict short-term mortality in patients with different causes of cirrhosis [28] and is now being used to predict waiting list mortality of patients listed for liver transplantation. The MELD score uses objective and standardized laboratory parameters (i.e., serum bilirubin, prothrombin time (international normalized ratio, INR), and creatinine) over a broader range of possible values to provide a more dynamic assessment of liver disease severity. Calculating the MELD score needs a calculator and cannot be easily done at the patient's bedside. 

### 5.2. Prevent Further Liver Damage

Alcohol, potentially hepatotoxic drugs including medications that may increase the risk of gastrointestinal bleeding (nonsteroidal anti-inflammatory agents), or renal insufficiency should be avoided. Patients with decompensated HBV cirrhosis should be vaccinated against hepatitis A if not already immune as superimposed hepatitis A infection could be fatal [29, 30]. As mentioned, the presence of HBeAg or HBV DNA indicates continuing viral replication. International guidelines suggest treating patients with chronic hepatitis B cirrhosis if serum HBV DNA present (EASL) or more than 2,000 IU/mL (AASLD/APASL). The threshold for antiviral therapy is usually lower for decompensated liver disease [31–33]. 

### 5.3. Prevent and Treat Complications of Cirrhosis

Gastroscopy should be performed on initial presentation and every two years afterwards in patients with liver cirrhosis to look for oesophageal and gastric varices [34]. If these are found, appropriate treatment should be instituted. Variceal bleeding can be prevented in grade 3 and 4 varices by oral beta blockers and endoscopicvariceal ligation [35]. Treatment of variceal bleeding should include antibiotics to prevent spontaneous septicaemia. Transjugular intrahepatic portosystemic stent (TIPS) placement may be required in patients with uncontrolled or recurrent variceal bleeding [36]. TIPS may also be considered in patients with refractory ascites if their liver function is not severely impaired, if they are less than 70 years old, and if hepatic encephalopathy is absent [37]. Spontaneous bacterial peritonitis (SBP) and other spontaneous infections should be treated straight away with broad spectrum antibiotics, (e.g., cephalosporins or amoxicillin/clavulanate) and albumin to prevent the hepatorenal syndrome [38]. Prophylactic antibiotics should be given to patients with a history of SBP [39]. 

Hepatic encephalopathy is a severe complication of cirrhosis and is related to the effect of ammonia. Recent evidence suggests that the effect of ammonia on the brain is triggered by inflammation caused by spontaneous infections. The mainstay of therapy is antibiotics (neomycin, rifaximin, vancomycin) and nonabsorbable disaccharides. Protein restriction is no longer recommended and can worsen the nutritional status if maintained [40]. The development of HE in patients with cirrhosis is associated with a less than 50% survival at 1 year. Liver transplant should be considered. Hepatorenal syndrome (HRS) is a potentially lethal complication and is usually triggered by infections. Besides antibiotics, it can be effectively treated with vasoconstrictors associated with intravenous albumin, TIPS, and albumin dialysis [41] .

### 5.4. HCC Surveillance

Patients should undergo HCC surveillance by determining serum alpha-fetoprotein (AFP) levels and liver ultrasound every 6 months [32] (Table 2). Early stage HCC can be successfully managed by loco-regional ablative therapy [42] and may change the priority for transplantation [43]. 

## 6. Liver Transplantation

Liver transplantation is a well-established modality for treating patients with advanced irreversible liver failure for which there are no alternative treatments [43]. Approximately 5% of liver transplants performed in the United States annually are for hepatitis B [44], and the proportion is higher in the Asia Pacific region [33]. All cirrhotic patients with a CTP score of more than 7 and a complication of portal hypertension such as ascites, encephalopathy, or variceal bleeding should be referred for liver transplant evaluation [43]. Selected patients with unresectable HCC that is less than 5 cm in maximal diameter should also be referred for liver transplant evaluation. 

Immunoprophylaxis using prolonged high-dose hepatitis B immunoglobulin (HBIG) has resulted in excellent patient and graft survival rates for patients with decompensated HBV cirrhosis who were viraemic pretransplant [5, 45]. Up to 40% of patients with pretransplant viraemia who received HBIG alone developed recurrent HBV infection [5]. This risk of posttransplant HBV recurrence can be reduced by antiviral suppression of HBV replication prior to transplantation and maintenance of antiviral therapy after transplantation [33]. Liver transplantation is not available to many patients with decompensated HBV infection in the Asia Pacific region, and the only recourse for these patients is antiviral therapy.

## 7. Antiviral Treatment

Suppression of HBV replication has resulted in reduction of hepatic necroinflammation and improvement of liver function in patients with CHB cirrhosis and liver decompensation. Patients with decompensated HBV-cirrhosis should be considered for antiviral therapy irrespective of HBVDNA levels. 

### 7.1. Interferon-Alpha

Interferon-alpha or its pegylated version is safe and effective in patients with chronic hepatitis B and in selected patients with compensated HBV cirrhosis [46, 47]. It has been associated with life-threatening hepatitis flares (up to 50%) and infectious complications (28%) in prospective trials of patients with decompensated HBV cirrhosis even when used in very low doses [48, 49]. It is generally discouraged in patients with decompensated HBV cirrhosis. 

### 7.2. Oral Antiviral Agents

Most practice guidelines recommend prescribing an oral nucleos(t)ide analogue (and not interferon) for patients with decompensated HBV cirrhosis independent of the patients serum ALT, HBV DNA level, and HBeAg status [31–33]. These recommendations are largely based upon open-label studies of lamivudine and adefovir in this group of patients. These studies reported that antiviral therapy was associated with improved outcomes including a delay or prevention in the need for liver transplantation (Table 1) [50–53]. A biphasic survival pattern was noted with most deaths occurring within the first 6 months of treatment; patients with higher pretreatment bilirubin, creatinine, and HBV DNA levels were at greatest risk for early death while early suppression of HBV replication was not associated with more favorable outcomes [51]. 

## 8. Lamivudine

Lamivudine is an orally administered nucleoside analogue that inhibits HBVDNA synthesis by incorporating active triphosphate (3TC-TP) into growing DNA chains. It suppressed serum HBV DNA to undetectable levels (using hybridization assays) in more than 90% of patients with compensated chronic hepatitis B. This was associated with improved serum ALT levels as well as liver histology at 12 months [54, 55]. It is generally safe and well tolerated with a side effect profile similar to that of placebo [54], making it the preferred treatment compared to IFN for patients with decompensated HBV cirrhosis. Hepatitis flares during treatment usually indicate the occurrence of resistant mutations. The recommended dose of lamivudine is 100 mg daily. Dose modification is necessary in renal impairment (reduction) and in patients with HIV coinfection (increment). Once treatment is initiated, it should be maintained indefinitely even in patients who appear to have dramatic clinical improvement and in those undergoing liver transplantation.

Development of lamivudine resistance begins after 6 months of treatment, and up to 70% of patients become lamivudine resistant after 5 years of continuous therapy [32]. Resistance to lamivudine is manifested by the reappearance of HBV DNA after its initial suppression with a variable increase in serum ALT levels [55, 56]. The most common mutation involves the YMDD motif of the HBV polymerase gene (M204V/I) and is frequently accompanied by another mutation in an upstream region (L180M) [57]. Diagnostic assays for lamivudine-resistant mutants are commercially available. Hepatitis flares are not uncommon with the emergence of YMDD mutants resulting in progressive worsening of liver disease [58, 59] and can be fatal in patients with decompensated disease.

Lamivudine resulted in a rapid suppression of HBV DNA to undetectable levels (non-PCR-based assays) and improvement in biochemical and clinical parameters in both controlled and uncontrolled studies of patients with decompensated HBV cirrhosis [51, 60–65]. Twenty-three out of 35 decompensated HBV patients treated by Villeneuve and colleagues showed a slow but marked improvement in biochemical parameters and CTP scores [60]. Seven patients underwent liver transplantation, and 5 patients died within the first 6 months of lamivudine treatment. Two of these 23 patients later perished (from SBP and HCC, resp.) and 3 developed lamivudine resistance. 

Significant improvement in CTP scores (8.3 versus 6.7) and ALT levels (111 versus 58 IU/L) were also noted in 18 Indians with decompensated HBV cirrhosis after a mean treatment duration of 18 months using lamivudine [61]. Yao and Bass reported similar CTP score improvement in 13 patients with Child's C cirrhosis given lamivudine and 5 of the patients were eventually taken off the liver transplant waiting list [62]. Similar findings were also noted in 30 Greek patients with decompensated HBV cirrhosis given lamivudine [63].

More than 80% of 154 patients with decompensated HBV cirrhosis had suppression of HBV DNA to undetectable levels by the branched-chain DNA (bDNA) assay within 8 weeks of initiating lamivudine treatment by Fontana and coworkers [51]. HBeAg loss was seen in 35% patients and HBeAg seroconversion to anti-HBe occurred in 20% of patients. The actuarial 3-year survival was 72% for all patients and 88% for patients who survived beyond the first 6 months of treatment.

In a study involving 77 HBsAg-positive liver transplant candidates, Perrillo et al. reported stabilization or improvement in liver disease severity with lamivudine in 27 patients without transplants who were treated with lamivudine for a median of 28 months [64]. The actuarial survival in these patients appeared to be better than the survival in untreated historical controls with decompensated HBV cirrhosis and similar to that of patients with untreated compensated HBV cirrhosis from an earlier observation [3]. 

Yao et al. noted that transplant candidates receiving lamivudine were less likely to undergo transplantation than untreated historical controls who were matched for age, gender, and illness severity at the time of listing (35% versus 74%, *P* = .04) [65]. A significantly greater proportion of the lamivudine-treated patients experienced an improvement ≥3 points in their CTP scores compared with the untreated historical controls (61% versus 0%, *P* = .0001). In a retrospective analysis of 309 North American HBsAg positive liver transplant candidates, Fontana and his colleagues compared the outcomes of 162 lamivudine-treated patients and 147 untreated patients [66]. The two groups were comparable in liver disease severity before treatment. Treated patientswere more likely to have evidence of active HBV replication. Overall, the actuarial pretransplant and transplant-free survival was similar in the two groups and lamivudine had no apparent effect on liver disease severity in patients who underwent transplantation. However, among the patients who were still awaiting transplantation, lamivudine appeared to stabilize or improve liver disease severity.

Earlier studies using lamivudine in decompensated HBV cirrhosis were not controlled, and control cohorts used in later studies were either historical or non-randomised. Inclusion criteria and therapeutic endpoints were also inhomogeneous. It was unclear whether patients in some of the studies had acute hepatic decompensation secondary to a recent hepatitis flare as improvement upon viral suppression is more likely in group than in patients with hepatic decompensation secondary to end-stage liver disease [60, 61]. Other interventions that may have prolonged transplant-free survival (e.g., TIPS, use of prophylactic antibiotics) may have contributed to the observed improvements in clinical outcomes. In spite of all these inadequacies, lamivudine was found to be safe in patients with decompensated HBV cirrhosis although not all patients benefited from it [67]. Clinical improvement usually occurs between 3 to 6 months of therapy and improvement might not occur if treatment is started late. Pretreatment severity of liver disease (increased bilirubin, low albumin, prolonged PT and raised creatinine) is a more important predictor of early mortality than antiviral response in this group of patients [51, 66]. Careful monitoring is mandatory in patients with decompensated liver disease treated with lamivudine as a hepatitis flare from resistant mutants can be fatal. Should molecular resistance be detected add on therapy with adefovir dipivoxil or substitution therapy with tenofovir or entecavir is advised. Patients with initial clinical improvement can develop complications of cirrhosis and HCC even in the absence of lamivudine-resistance. 

## 9. Adefovir Dipivoxil

Adefovir dipivoxil is a prodrug of adefovir, an acyclic nucleotide analog of adenosine monophosphate. Adefovir is phosphorylated to the active metabolite, adefovir diphosphate, by cellular kinases. Adefovir diphosphate inhibits HBV DNA polymerase (reverse transcriptase) by competing with the natural substrate deoxyadenosine triphosphate and by causing DNA chain termination after its incorporation into viral DNA [33]. It has a high genetic barrier to resistance and has the ability to suppress most lamivudine-resistant mutants. Renal toxicity is rare with the dose of 10 mg daily (Table 3). Adefovir dipivoxil has been available for the treatment of chronic hepatitis B since 2003 but has not been evaluated as a primary treatment for patients with decompensated cirrhosis.

In a compassionate use study involving 128 patients with decompensated cirrhosis and 196 patients with recurrent hepatitis B after liver transplant, addition of adefovir resulted in a 3-4 log10 reduction in serum HBVDNA levels, which was sustained throughout the course of treatment [52]. After 48 weeks of treatment, undetectable HBV DNA by PCR and normal ALT was noted in 81% and 76% of the pretransplant and 34% and 49% of the posttransplant patients, respectively. More than 90% of the pretransplant patients had improvement in their CTP scores, and 1-year survival was 84% for the pre- and 93% for the posttransplant patients. Follow-up data on 226 pretransplant patients showed that viral suppression was maintained in 65% of patients after 96 weeks of treatment with accompanying improvement in CTP and MELD scores. Fourteen percent of patients died within the first year and at least 33% required liver transplantation for long-term survival [53].

A recent interim report showed no difference in mortality rates after 24 weeks of treatment in 195 patients with decompensated HBV cirrhosis randomized to adefovir or entecavir. This study is in progress [68]. 

Although antiviral drug resistance is substantially less common with adefovir monotherapy compared to lamivudine, concerns remain regarding the slow rate of suppressing HBV replication with adefovir as well as the potential for dose-dependent nephrotoxicity in decompensated HBV patients (up to 28% of patients had an increase in serum creatinine ≥0.5 mg/dL after 48 weeks of treatment [69, 70]. Until more data becomes available, adefovir should not be recommended as first-line treatment in patients with decompensated HBV-cirrhosis. However, for patients with worsening liver disease secondary to lamivudine resistance, use of adefovir as a salvage therapy is an option.

## 10. Telbivudine

Telbivudine, a synthetic thymidine nucleoside analogue, is active against HBV. It undergoes phosphorylation by cellular enzymes to form the active metabolite, telbivudine triphosphate which incorporates into viral DNA competing with the natural substrate, thymidine triphosphate, and causing DNA chain termination, resulting in inhibition of HBV replication. It has demonstrated potent activity against hepatitis B with a significantly higher rate of response and superior viral suppression compared with lamivudine and adefovir [71]. It is generally well tolerated with a low adverse effect. It was approved by the FDA in late 2006. HBV strains with reduced susceptibility to telbivudine have emerged during therapy with the drug. Cross-resistance may occur among some nucleoside analogues active against HBV. Lamivudine-resistant HBV with reduced susceptibility to telbivudine has been observed. Some adefovir-resistant HBV are also resistant to telbivudine. 

Gane and colleagues conducted a double blind trial on 195 patients (70% Asians) with decompensated HBV liver disease [72]. Patients were randomly assigned to receive 600 mg telbivudine or 100 mg lamivudine for 104 weeks. About three-quarters were men, with a mean age of 52 years and 57% were HBeAg negative. In a 2-year intent-to-treat analysis, more patients appeared to have undetectable HBV DNA (<300 copies/mL; 47% versus 36%, *P* = .15) and ALT normalization (58% versus 50%, *P* = .25) in the telbivudine treatment arm than in the lamivudine arm. Using a composite endpoint of undetectable HBV DNA and ALT normalization, however, telbivudine performed significantly better than lamivudine (34% versus 24%, resp.; *P* = .004). 29% of telbivudine recipients experienced viral breakthrough while on therapy, compared with 39% of lamivudine recipients (*P* = .16). At the end of treatment, about 75% of patients in both arms had stabilized or improved liver disease, as indicated by changes from baseline in CTP scores. Kidney function (indicated by glomerular filtration rate) modestly improved in the telbivudine arm, while worsening in the lamivudine arm. Early (week 24) survival rates were similar in the 2 study arms, 96% with telbivudine and 92% with lamivudine. Long-term (week 104) survival rates were 96% and 83%, respectively, with a trend toward statistical significance. Serious adverse events were common, consistent with advanced liver disease, and they occurred with similar frequency in both arms (55% of telbivudine recipients versus 61% of lamivudine recipients). No cases of rhabdomyolysis or lactic acidosis were reported. The investigators concluded that telbivudine was well tolerated with stabilization of liver function and had comparable tolerability to lamivudine. 

## 11. Entecavir

Entecavir is a cyclopentyl guanosine analogue with potent selective inhibition of the priming, DNA-dependent synthesis, and reverse transcription functions of HBV polymerase. It has demonstrated activity against both wildtype HBV and, to a lesser extent, lamivudine-resistant HBV [73, 74]. It suppresses HBV replication more rapidly and effectively than lamivudine or adefovir in patients with compensated chronic HBV [75, 76]. It has an excellent resistance profile after 5 years in nuycleosid(t)e naïve patients and does not have any reported nephrotoxicity [77], It has been used to rescue a small number of liver transplant recipients with lamivudine-resistant HBV successfully [78].

Shim et al. demonstrated that 0.5 mg entecavir daily was effective in treating 70 nucleoside naïve decompensated HBV patients with nearly 90% achieving undetectable HBV DNA (PCR) at 1 year [79]. The virological responses in 55 decompensated HBV patients treated for at least 1 year were compared to 144 compensated patients treated with entecavir from the same center. The mean MELD (11.5 versus 7) and CTP scores (8.1 versus 5.3) were significantly higher in the decompensated patients. The proportion of HBeAg positive patients and mean HBV DNA levels were similar in the two groups. Overall, the 1-year transplant-free survival rate was 87% in the decompensated patients. As seen previously with lamivudine, the majority of adverse outcomes occurred during the first 6 months of therapy with the nine patients having more severe liver failure at entry. Baseline HBV DNA levels, HBe antigenaemia and response to therapy were similar in both survivors and non-survivors or those who underwent transplant. Nearly 50% of the entecavir treated patients had a clinically significant decrease in their CTP score of >2 points at 1-year. HBeAg loss in both the decompensated and compensated patients was remarkably high at 1 year (48% vs 41%). HBV DNA suppression was maintained during followup with no instances of viral rebound or entecavir-resistant HBV. Not all decompensated patients improved with entecavir therapy. Twelve patients (22%) showed no change in their CTP score at 1-year (4 patients had aggravation or their liver disease with worsening CTP scores). Five patients developed HCC during followup.

A retrospective analysis of 107 decompensated patients (mean age 53 years; 70.1% men; 42% HBeAg positive) treated with lamivudine or entecavir showed significantly lower serum HBV DNA levels and prevalence of patients with undetectable HBV DNA (PCR) at 3, 6, 9, and 12 months after treatment in entecavir-treated patients than in the lamivudine group. Serum ALT levels, CTP and MELD score, and the prevalence of patients with improved CTP scores at 3, 6, 9, and 12 months did not differ between two groups. The prevalence of HBeAg seroconversion and HCC and mortality also did not differ between two groups while that of viral breakthrough was significantly more frequent in the lamivudine-treated patients [80].

Liaw et al. randomized 195 patients with decompensated HBV to entecavir (1.0 mg per day) or aderovir (10 mg per day) [68], One-third (34%) of patients had lamivudine-resistant HBV. Interim results at week 24 demonstrated a significantly greater reduction in HBV DNA and serum ALT levels in the entecavir treated patients. The 24 week mortality rates were similar in both treatment arms. Entecavir was well tolerated and safety results were comparable in both treatment groups. Continued followup is needed since the rate of entecavir-resistant HBV can substantially increase over time in lamivudine-resistant HBV infection and potentially fatal flares may develop [81].

Entecavir was recently compared with tenofovir + emtricitabine combination and tenofovir singly in an ongoing multicentre study [82]. Improvements in CTP and MELD scores as well as frequency of undetectable HBV DNA at week 48 were similar in the three treatment arms. 

Severe lactic acidosis with entecavir has been reported in patients with decompensated liver [83]. 

## 12. Tenofovir

Tenofovir is an acyclic nucleotide analog with a molecular structure similar to that of adefovir. It is approved for the treatment of HIV infection and has in vitro activity against both wild type and lamivudine-resistant HBV [84]. It is administered as the prodrug tenofovir disoproxil fumarate (TDF), and it is converted to tenofovir by plasmaesterases. Tenofovir is phosphorylated to the active metabolite which works as a chain terminator if incorporated into the DNA chain and is a competitive inhibitor of natural deoxyadenosine 50-triphosphate. Tenofovir is eliminated by a combination of glomerular filtration and active tubular secretion. It is a significantly more potent suppressor of HBV replication than adefovir and no drug-resistant variants have been reported with 4 years of continuous treatment in compensated HBV patients [85]. 

In the ongoing study reported in the previous section [84], on 112 decompensated HBV patients given tenofovir, tenofovir + emtricitabine, or entecavir, there were more undetectable HBV DNA at week 48 in the tenofovir containing treatment arms (71%) than in the entecavir treatment arm (33%) in patients with lamivudine resistant HBV. HBeAg seroconversion was seen in 21 % and 13% of the tenofovir and tenofovir/emtricitabine arms, respectively, but not in the entecavir arm. Rates of nephrotoxicity, tolerability and patient mortality were similar in the three treatment arms through week 48. Continued follow-up of these patients is needed to determine which of the newer antiviral agents can offer the best risk-benefit ratio in this challenging patient population. 

Although a tenofovir-based regimen may be preferred in decompensated patients with lamivudine-resistant HBV, there are concerns regarding the long-term safety of tenofovirin some HBV patients including nephrotoxicity and metabolic bone disease [86, 87]. Patients with decompensated cirrhosis are frequently malnourished and may have low vitamin D levels. Prospective studies of bone density and metabolic parameters during prolonged tenofovir treatment are warranted as well as potential calcium and vitamin D supplementation [88]. 

## 13. Summary

The availability of safe, orally administered antiviral agents has revolutionized the management of chronic HBV and opened up new treatment options for the large number of patients with decompensated HBV cirrhosis worldwide who previously had a dismal prognosis. These drugs can improve or stabilize liver disease in patients who are not transplant candidates or have no access to liver transplantation. For these patients, the oral HBV antivirals may represent the only hope for better quality or longer duration of survival and reduced utilization of health care resources. The aim of treatment in transplant candidates is to improve their functional status such that they eventually might be removed from the transplantation list. All patients with decompensated cirrhosis, regardless of their serum HBV DNA level, should be considered for treatment. Decompensated patients with evidence of active HBV replication (i.e., presence of HBe antigenemia and HBV DNA > 2000 iu/mL,) are more likely to derive benefit from antiviral therapy. 

Clinical studies have confirmed that oral antivirals are generally safe and effective in suppressing HBV replication in decompensated HBV cirrhosis with resultant stabilization or improvement in liver disease. Clinical improvement is slow and takes 3 to 6 months. It is not certain if starting treatment earlier will improve the rate of response. Recent efficacy and safety data supports the use of entecavir as a first-line treatment option for nucleos(t)ide naive patients with decompensated HBV cirrhosis [89]. Lamivudine and telbivudine are also safe agents, but the risk of resistance with prolonged therapy is ever present with potential for worsening liver disease and increased risk of HBV recurrence after transplantation and vigilance is important. Tenofovir or entecavir monotherapy or adefovir add-on therapy are possible rescue options should resistance occur. Studies evaluating tenofovir monotherapy and combination therapy in patients with decompensated cirrhosis are in progress. However, continued follow-up from these ongoing studies including long-term efficacy, safety, and resistance data are needed. Further studies are also needed to identify the optimal agent(s) for patients with decompensated lamivudine-resistant HBV cirrhosis. Decompensated HBV patients receiving oral nucleos(t)lde analogues must undergo frequent clinical and laboratory assessment to insure medication compliance and surveillance for vIrological and clinical response as well as drug side effects, drug resistance, and HCC. As it is not possible to identify which patients with high CTP or MELD scores will have poor short term prognosis, it is advisable to refer all decompensated HBV patients for liver transplant evaluation at presentation if available.

Antiviral therapy should be given to all potential liver transplant candidates with decompensated HBV cirrhosis and detectable HBV-DNA. Lamivudine resistance will result in HBV recurrence in the posttransplant period [33]. Adefovir and entecavir can be given to rescue lamivudine resistance, and initial use of these agents may minimize drug resistance. Lamivudine plus low-dose intramuscular HBIg (400–800 U daily for 1 week, then monthly) is as effective as lamivudine plus high-dose intravenous HBIg in preventing recurrent HBV infection resulting in a 5-year graft survival of up to 85% at 10% of the cost [90]. Substituting HBIg with adefovir 12 months posttransplant also prevent late HBV recurrence and costs much less [91]. Lamivudine plus adefovir combination from the time of listing has been shown to be well tolerated, prevent lamivudine resistance prior to transplant, rescued some patients from the need for transplantation, and prevented recurrent HBV infection following liver transplantation, regardless of baseline HBV-DNA status [92]. Patients who were HBV-DNA negative prior to transplant and those with sustained protective levels of anti-HBs following posttransplant vaccination can be safely given lamivudine or entecavir monotherapy 12 months after transplant. Antiviral prophylaxis should also be given in patients who have received an anti-HBc(+) liver to prevent de novo HBV infection. 

Although the outlook for decompensated HBV patients is bright with the advent of these oral antivirals, emphasis should be placed on effective treatment of patients with chronic HBV infection to prevent them from progressing to the decompensated state. 

## Figures and Tables

**Table 1:** Assessing liver disease severity in decompensated HBV cirrhosis.

Scale (range)	Mild	Moderate	Severe	Ref.
CTP (5 to 15)	5-6 (A)	7–9 (B)	10–15 (C)	Keeffe, 2001 [43]
MELD (6–40 )	6–10	11–24	25–40	Kamath et al., 2001 [28]

**Table 2:** General Recommendations in Decompensated HBV Cirrhosis.

Assess disease severity	Clinical, liver biochemistry, creatinine, INR CTP score, MELD score
Prevent further liver damage	Avoid alcohol
	Avoid hepatotoxic drugs
	Avoid Immunosuppression. Antiviral prophylaxis if necessary
	Avoid Aspirin/NSAIDS
	Hepatitis A vaccination in nonimmune

Prevent and treat	Laboratory and clinical assessment 3 to 6 monthly

Complications	Endoscopy at presentation and treat varices accordingly
	Be aware of spontaneous infections and treat appropriately
	Salt and fluid restriction in ascites control, TIPS
	Albumin and terlipressin in hepatorenal syndrome
	Antibiotics and nonabsorbable disaccharides in hepatic encephalopathy
	Low-protein diet not essential
	Regular AFP measurement and ultrasound examination

Antiviral therapy	Entecavir
	Lamivudine. Replace with entecavir monotherapy, Tenofovir monotherapy, or add on adefovir in cases of lamivudine resistance
	Tenofovir
	Telbivudine
	Adefovir

Liver transplantation	Pretransplant antiviral therapy in viraemic subjects and immunoprophylaxis using HBIG after transplant

**Table 3:** Antiviral Agents with Activity against Wild Type and Lamivudine resistant HBV.

Agent	Daily dose	Side effects	Comments
Adefovir	10 mg	Dose-dependant nephrotoxicity	Drug resistance after 12 months
Entecavir	1 mg	No major side effects to date	Drug resistance eventually in lamivudine-resistant mutants
Tenofovir	300 mg	Neuropathy, nausea, CPK elevations, Fanconi syndrome	No drug resistance up to 4 years
